# Strengthening tRansparent reporting of reseArch on uNfinished nursing CARE: The RANCARE guideline

**DOI:** 10.1002/nur.22103

**Published:** 2021-01-02

**Authors:** Catherine Blatter, Patti Hamilton, Stefanie Bachnick, Franziska Zúñiga, Dietmar Ausserhofer, Michael Simon, for the RANCARE Consortium

**Affiliations:** ^1^ Institute of Nursing Science University of Basel Basel Switzerland; ^2^ College of Nursing Texas Woman's University Denton Texas USA; ^3^ College of Health Care‐Professions Claudiana Bozen Italy; ^4^ Nursing Research Unit Inselspital Bern University Hospital Bern Switzerland

**Keywords:** nursing research, reporting guideline, transparency, unfinished nursing care

## Abstract

Unfinished, rationed, missed, or otherwise undone nursing care is a phenomenon observed across health‐care settings worldwide. Irrespective of differing terminology, it has repeatedly been linked to adverse outcomes for both patients and nursing staff. With growing numbers of publications on the topic, scholars have acknowledged persistent barriers to meaningful comparison across studies, settings, and health‐care systems. The aim of this study was thus to develop a guideline to strengthen transparent reporting in research on unfinished nursing care. An international four‐person steering group led a consensus process including a two‐round online Delphi survey and a workshop with 38 international experts. The study was embedded in the RANCARE COST Action: Rationing Missed Nursing Care: An international and multidimensional Problem. Participation was voluntary. The resulting 40‐item RANCARE guideline provides recommendations for transparent and comprehensive reporting on unfinished nursing care regarding conceptualization, measurement, contextual information, and data analyses. By increasing the transparency and comprehensiveness in reporting of studies on unfinished nursing care, the RANCARE guideline supports efficient use of the research results, for example, allowing researchers and nurses to take purposeful actions, with the goal of improving the safety and quality of health‐care services.

## 1 INTRODUCTION

Incomplete or unfinished nursing care has repeatedly been associated with adverse outcomes for patients (e.g., increased rates of mortality, falls, and nosocomial infections), nurses (e.g., higher burnout and lower job satisfaction) and health‐care organizations (e.g., higher turnover), thus emphasizing its significance in health services research (Jones et al., 2015; Recio‐Saucedo et al., 2018). A growing body of international literature from diverse settings allows us to map the nature and extent of unfinished nursing care. Up to 98% of nurses working in hospital inpatient care report having left necessary care activities unfinished or undone during recent shifts (Jones et al., 2015; Recio‐Saucedo et al., 2018). Across various settings, the activities reported left unfinished most often include emotional support, patient education, coordination, and discharge planning and care planning. Yet, meaningful comparison between studies is hindered by the phenomenon's complexity, paired with a lack of transparency and comprehensiveness in reporting (e.g., on care task list, scoring cutoffs, or handling of missing values) and lack of consistency of terminology (e.g., use of different terms, care left undone, and missed nursing care, for the same instrument [cf. Ausserhofer et al., 2014; Ball et al., 2018]).

## 2 BACKGROUND

Unfinished nursing care is “a problem of time scarcity that precipitates the process of implicit rationing through clinical priority setting among nursing staff resulting in the outcome of care left undone” (Jones et al., 2015). Introduced by scholars as an umbrella term, it covers multiple closely related, partly overlapping phenomena found in the literature often referred to as *missed care* (Kalisch & Williams, 2009), *implicit rationing of nursing care* (Schubert et al., 2007), *care or tasks left undone* (Aiken et al., 2001), or *omitted nursing care* (Vincelette et al., 2019). In accordance with previous research (Jones et al., 2019; RANCARE COST‐Action 15208 & EU‐COST Association, 2016) and to ensure inclusivity of all related concepts, we refer to *unfinished nursing care* throughout this article.

The quantity of unfinished nursing care is traditionally measured using multi‐item scales in self‐report surveys filled out (mostly) by nursing staff. To address some of the known limitations of self‐reported measures, more novel approaches have recently been reported, such as the use of a single‐item measure (Hamilton et al., 2017) or identification of missed care activities through the use of data from electronic health records (Dall'Ora et al., 2019).

While the research community now widely acknowledges the common underlying phenomenon of unfinished nursing care, differences in its key elements are still reflected in publications: quantitative studies on the topic face a variety of methodological and conceptual challenges (Vincelette et al., 2019) that are thus reflected in measurement and analyses as well as the transparency and comprehensiveness of their reporting. High levels of variability are seen not only between studies investigating different conceptualizations (e.g., missed care vs. implicit rationing) but also within studies using a single concept and corresponding instrument (e.g., different cutoffs in the scoring of multi‐item surveys; different terminology used for the same instrument and sample; differing task list within the same approach [tasks undone scales TU‐5 and TU‐13]; differing temporal frame for the reference period [previous 7 working days vs. previous 30 days vs. previous 7 shifts worked]; differing aggregation, on individual, unit‐level, or institution level). The lack of transparency and comprehensiveness on these elements complicates interpretation and meaningful comparison of study findings enormously.

In February of 2017, members of the European RANCARE 15208 COST Action titled *“*RATIONING–MISSED CARE: An international and multidimensional problem (RANCARE)*”*—a 4‐year international networking activity aiming to advance a cross‐national discussion on unfinished nursing care with implications for practice, professional development, and education (RANCARE COST‐Action 15208 & EU‐COST Association, 2016) emphasized the need to include key information in publications to allow improved assessment of reported studies' contributions to the wider body of knowledge regarding unfinished nursing care. To address such barriers for comparison and provide guidance for future research, this study's aim was to develop a guideline that would strengthen transparent reporting of quantitative research on unfinished nursing care.

## 3 METHODS

The Strengthening tRansparent reporting of reseArch on uNfinished nursing CARE (RANCARE) guideline was developed following steps outlined by the EQUATOR network (Enhancing the QUAlity and Transparency Of health Research) (Moher et al., 2010). An international four‐person steering group led a consensus process featuring a modified online Delphi survey to identify, refine, and agree on relevant items for the guideline (Hsu & Sandford, 2007). This group was comprised of three researchers experienced in the field (Patti Hamilton, Dietmar Ausserhofer, and Michael Simon) and a graduate student (Catherine Blatter). The steering group has experience in investigating the concepts rationing of care (Dietmar Ausserhofer and Michael Simon), nursing care left undone (Dietmar Ausserhofer), and missed care approaches (Patti Hamilton), as well as applying conceptual comparison (Patti Hamilton). To identify experts for the Delphi survey, we performed an unpublished update of the literature review on studies investigating unfinished nursing care from Jones et al. (2015), with extension to all settings. Participants were considered eligible for invitation if they were listed as first or corresponding authors on any of these papers. Second, all members of the RANCARE 15208 COST Action were considered eligible for participation. COST Actions are networking activities aiming to connect researchers from academia, industry, public and private sectors to drive innovation in a specific field. The COST Action is funded over a course of 4 years by the European Cooperation in Science and Technology (COST; https://www.cost.eu/). Lastly, the list of eligible participants was screened for by the authors and potentially extended by hand with a snowballing approach. All eligible participants were invited via email.

Participation in this study was voluntary; informed consent was obtained before each Delphi round. All collected data were treated as confidential and presented anonymously in subsequent rounds. In accordance with Swiss law, the responsible ethics committee exempted the Delphi study from its oversight (EKNZ, Req‐2018‐00399).

Between July 2017 and March 2019, the steering group screened the literature and convened several times to organize two online Delphi rounds and one face‐to‐face workshop. Based on a review of the literature on unfinished nursing care and a review of existing methodological guidelines, we developed a list of *n* = 61 items, capturing methodological or conceptual barriers in research on unfinished nursing care, which were introduced for feedback on their relevance (“How do you rate the relevance of this item for inclusion in the RANCARE guideline?”) and clarity (“How do you rate the clarity of wording of this item?”) in the Delphi survey. Rating of each item was made on a scale from 1* = extremely irrelevant/unclear* to 9* = extremely relevant/clear*, with a given middle of 5* = uncertain*. Consensus on items was defined a priori according to the RAND/UCLA Appropriateness Method (Fitch et al., 2001). In this method, the following measures are calculated for every item: median, interpercentile range (IPR; 30th−70th), asymmetry index (AI; absolute difference between central point of IPR and central point of measurement scale, i.e., 5), and IPR adjusted for asymmetry (IPRAS = 2.35 + (AI × 1.5)). Those are used to calculate the absolute disagreement index (DI = IPR/IPRAS). A DI < 1 indicates no extreme variation of answers, also stated as the absence of disagreement. Finally, consensus on relevance/clarity of an item is classified as the median score of an item lying within the 3‐point range of 7–9 in the absence of disagreement (Fitch et al., 2001; Van Grootven et al., 2018). Participants were invited to leave suggestions for improvement at any time via comments.

Of 126 eligible participants, 62 (return rate: 49%) completed the first Delphi round, with 38 (30%) eventually finishing both rounds. Twenty‐six also joined the face‐to‐face workshop. The majority of participants were females (76%) and had a background in nursing (70%) or psychology (11%), followed by educational sciences (5%), business and economics (5%) or midwifery, sociology and health systems (each 3%). Seventy‐five percentage of participants had more than 5 years' professional experience; 30% had >20 years. Eighty percentage held doctoral degrees, with two‐thirds holding positions as assistant, associate, or full professor.

The initial item list of *n* = 61 items reached consensus (i.e., median rating ≥7 without disagreement) regarding relevance and clarity on 98% of the items in the first Delphi round. Fifty percentage of participants provided additional feedback through comments. With respect to the comments' significance, the steering group agreed to adjust and refine the items for further discussion during the face‐to‐face workshop. The main feedback obtained both from Round 1 and during the workshop included removing redundant items, clarifying both the format and potential applications for qualitative research as well as incorporating examples.

Based on results from the first Delphi round and the workshop, we sent a list of 38 revised guideline items in Delphi Round 2 for rating on relevance (1–9), clarity (1–9) and we added the self‐developed question, whether the item should be included (yes/no), to allow for a clear decision. For each item, the median rating was ≥7 without disagreement on either relevance or clarity and therefore included in the guideline. Furthermore, 17 (45%) items were rated by >90% of participants as to be included. The remaining 21 (55%) items were rated for inclusion by ≥75% of participants. With all items' acceptance confirmed, the Delphi survey was terminated. Substantial comment‐based feedback was implemented (namely, clarification of wording and splitting two items), resulting in the final version of the guideline.

## 4 RESULTS

The components of the final RANCARE guideline consist of 40 items that address key elements influencing the reporting of quantitative research on unfinished nursing care (cf. Table 1). It is structured according to common sections of a research paper. Many items are self‐explanatory and applicable to most observational research; others target primarily self‐report measures or routinely collected data.

**Table 1:** The RANCARE guideline

RANCARE guideline—strengthening tRansparent reporting of reseArch on uNfinished nursing CARE
Please consider the following points when using the RANCARE guideline:
• RANCARE guideline provides guidance for transparent reporting of quantitative research on unfinished nursing care.
• Unfinished nursing care is used as an umbrella term throughout this guideline for all commonly used concepts related to the topic (e.g., missed care, implicit rationing, task undone, care left undone, care omitted).
• The guideline addresses issues commonly encountered in communicating quantitative research on the topic—however, some aspects may be pertinent for qualitative or mixed‐methods research.
• Please use the RANCARE in addition to the reporting guideline relevant to your study's design (e.g., STROBE or CONSORT)
• All items are offered as *points to consider*—they may not be applicable to every study or need to be left out due to limited space for publications.
Section 1: Title/Introduction/Background
(1) Title
(a) Indicate the term for the concept used in the study to represent the phenomenon of unfinished nursing care
(2) Keywords
(a) In addition to the term of your key concept, include “unfinished nursing care” as keyword
(3) Background
(a) Provide a comprehensive literature review about your chosen term and its relationship to the broader field of unfinished nursing care
(b) Explain how your study findings will advance the state of science on unfinished nursing care
(4) Conceptual model/Theoretical consideration
(a) Describe the theoretical perspective/paradigm concerning unfinished nursing care underlying your study
(b) Where appropriate, include a figure describing the relationships between unfinished nursing care and other concepts to be studied
(5) Objectives
(a) State clearly the aims related to unfinished nursing care
Section 2: Methodology
(6) Study design
(a) Specify the study design in relation to the measurement of unfinished nursing care
(7) Setting/Context
(a) Provide relevant information about the context in which you are studying unfinished nursing care
(8) Sample/Respondents/Participants
(a) Describe the characteristics of study participants (e.g., nurses and patients)
(b) Specify inclusion and exclusion criteria of participants and/or data sources
(9) Sample/Data
(a) State the purpose of data collection
(b) Acknowledge if the data described in the paper were analyzed and reported in previous publications. If so, describe.
(10) Ethical and legal considerations
(a) Describe relevant ethical issues and how they were addressed (e.g., confidentiality for patients, respondents, records, and institutions)
(b) Include statement about ethical board review and approval
(11) Measurement of unfinished nursing care
(a) Indicate the method(s) for data collection (e.g., online survey, paper collection, existing records)
(b) State the instrument or item(s) for measuring unfinished nursing care
(c) Report all other methods or sources used (e.g., observation and electronic health records)
(d) *If multiple data sources were used*: clearly specify which data were used to answer which research question(s)
(12) Modification of a survey instrument *(if applicable)*
(a) Describe any modification on the original instrument that has been made (e.g., number and content of items, answer options) including the rationale for the modifications (e.g., setting, cultural and/or language adaption, skill level)
(b) Report any evidence on validity and reliability of the original and modified versions
(13) Time period/reference
(a) *For surveys:* Report the recall period for the self‐report of unfinished nursing care (e.g., last 7 working days, last shift, not specified)
(b) Clearly state the time frame referred to by the unfinished nursing care measure and other outcomes under study (e.g., unfinished nursing care measured per week and outcome data per month)
(14) Scoring
(a) Report how the scoring was made (e.g., mean score, sum score, percentage) for descriptive and inferential analyses and if any item weighting was made
(b) *If applicable:* specify at what level the data were aggregated (e.g., individual nurse/patient, unit, or facility level) and provide (statistical) justification for aggregation
(15) Statistical methods
(a) Explicitly link each statistical analysis to the relevant study aim
(b) Describe, in detail, statistical methods applied to analyze and model unfinished nursing care
(c) Describe adjustments for hierarchical data structure /clustering
(d) Specify any sensitivity or subgroup analysis
(e) State how missing data have been handled (e.g., was the case omitted from further analyses, missing values imputed)
Section 3: Results
(16) Descriptive results
(a) Provide relevant descriptive results on setting and context (Topic 7) as well as sample/respondents (Topic 8)
(17) Unfinished nursing care
(a) Report descriptive data for unfinished nursing care on the item level and aggregated for the overall score
(b) *If applicable*: Report unfinished nursing care for subgroups that are of interest for the study (e.g., nurses’ educational/leadership level)
(c) Report rates of missing data per item/subgroup/source
(d) *If applicable:* report hospital/facility and/or unit variability with a commonly used measurement (e.g., intraclass correlations)
Section 4: Discussion
(18) Interpretation & Implications
(a) Relate your results back to the original conceptual model and the theory used
(b) Discuss your results in the context of the larger body of knowledge of unfinished nursing care
(c) Discuss the implications for clinical practice, further research, education, and policy (depending on the journals’ focus and scope)
(19) Strengths & Limitations
(a) Report steps taken to reduce bias and confounding related to your study on unfinished nursing care
(b) Discuss limitations linked to your study on unfinished nursing care

The RANCARE guideline on Section 1 addresses the key aspect of conceptualization and terminology within the overall phenomenon of unfinished nursing care. Researchers should use the terminology of the concept studied throughout their paper (e.g., Item 1) but we recommend that they link their work to the umbrella term by including “unfinished nursing care” additionally as a keyword (Item 2). Adding this explicit link will facilitate literature searches and cross‐concept comparison. Furthermore, we encourage researchers to explore their research questions beyond the range of their terminology and refer to the overarching phenomenon of unfinished nursing care (Item 3). Still, authors should include a clear theoretical perspective of the paradigm underlying their study (Item 4). If necessary, this may be accompanied by an explanation of how the concept studied relates to unfinished nursing care. Both Items (3) and (4) are useful to embed the study in the research literature and to clarify assumptions and data generation processes.

The Section 2 addresses the methodology, including the transparent and comprehensive reporting of measurement and analyses of unfinished nursing care, as well as relevant contextual information. The latter (Item 7) should be applied at the system, organizational and individual levels, including comprehensive information about the setting's nursing staff and patient population, in addition to the sample description (Item 8). It is crucial to collect and report information on the context (e.g., workload and weekday/weekend) in which the individual nurse worked during the reference period as it impacts their priority setting and decision making and therefore the level of unfinished nursing care. Table 2 provides corresponding examples from system to individual level.

**Table 2:** Contextual elements to describe the setting in studies on unfinished nursing care

Level	Examples
System level	• Country including cultural traditions
	• City including population size
	• Health‐care system within country (e.g., funding system, for profit)
	• Scope of practice of nursing staff
Organizational/Provider level	• Setting (e.g., hospital, nursing home, and home care) and specific area within setting (e.g., ICU and dementia care units)
	• Size/volume of the facilities/units (no. of beds), classifications (e.g., Magnet® status), ownership (e.g., public)
	• Model of care (e.g., patient allocation, primary nursing, team nursing, lean management) or describe task allocation between (nursing) staff
	• Skill/grade mix on unit
Individual level^a^	• Characteristics of nursing staff (e.g., age, gender, and level of education)
	• Characteristics of patient population (e.g., age, gender, primary, and relevant ancillary diagnoses)
	• Information on shift, weekday (weekends), months of data collection

Abbreviation: ICU, intensive care unit.

a This information should be included independently from the sample description.

Another focus (targeting Items 11, 12, 13, and 14) is set on the estimate of unfinished nursing care. Researchers should describe their measurement in as much detail as possible. This should include information on the scoring method applied as well as justification for aggregating values to a higher level; survey studies should clearly describe the recall periods (Jones et al., 2016). Item 15 provides suggestions to describe the statistical analyses performed and account for the common characteristic of clustered data (e.g., nurses within units within hospitals). Given the variety of studies on unfinished nursing care, an increase in reporting transparency regarding setting, measurement, and analyses is important as this will allow for meaningful comparison across diverse health‐care systems and eventually meta‐analyses (De Leeuw & Hox, 2003).

The Section 3 encourages the comprehensive reporting of descriptive data for both contextual elements and sample (Item 16), as well as for estimates of unfinished nursing care (Item 17). As much information as possible should be provided either in the manuscript or as Supporting Information Material. The results for unfinished nursing care should include a total score to allow for raw comparison across studies. Jones et al. (2015) recommend reporting estimates as mean scores or % positive responses. Reporting descriptive item‐level findings, including missing data, as well as subgroups of interest (e.g., educational or leadership level) will improve understandability within specific contexts.

The Section 4 re‐emphasizes the link between the concept under study and the overarching concept of unfinished nursing care (Item 18). For conclusions to be meaningful and compelling, they should be discussed in light of findings from studies using different conceptualizations. If relevant results from mixed‐methods research are available, it is recommended that these be integrated to deepen interpretation possibilities. The RANCARE guideline also encourages critical reflection on the measurement of unfinished nursing care and approval of actions taken to reduce bias in this study (Item 19).

## 5 DISCUSSION

Regarding the reporting of quantitative research targeting unfinished nursing care, the RANCARE guideline addresses the key elements of conceptualization, measurement, data analyses, and presentation of study findings. It increases the potential for research on this phenomenon by facilitating comparison between studies investigating closely related concepts. To maximize its usability across multiple study designs, the RANCARE guideline overlaps with elements of other guidelines; therefore, we recommend using it in addition to any design‐focused reporting guidelines (e.g., STROBE and CONSORT).

With 446 entries currently listed in the EQUATOR network library for reporting guidelines (December 14, 2020), there is arguably a need to justify the development of yet another guideline. In recent years, reporting guidelines have increasingly been developed to improve the reporting of content‐related aspects for specific research fields, rather than just targeting a study design. For instance, the TIDieR‐checklist extends CONSORT (Consolidated Standards of Reporting Trials) for improved reporting of interventions (Hoffmann et al., 2014). In medical research, a recent example includes the RECOvER‐checklist that aims to improve reporting of ERAS (enhanced recovery after surgery)‐related studies (Elias et al., 2019). To date, most published quantitative research on unfinished nursing care has applied cross‐sectional designs and followed STROBE (STrengthening the Reporting of OBservational studies in Epidemiology) reporting guidelines. The RANCARE guideline augments and enhances the existing guidelines by addressing key elements of research on unfinished nursing care such as conceptualization, corresponding measurement, and data analyses. Due to the complexity of the phenomenon we refrained from limiting the RANCARE guideline to specific sections and instead explicitly phrased all sections irrespective of the possible duplication with the corresponding design‐oriented guideline. We are well aware that not all sections may be applicable for each study but nonetheless understand our work as a collection of information needed for comprehensive reporting and to assess a study's quality. The impact of unfinished nursing care on health‐care delivery justifies a focused effort to deepen understanding by encouraging efficient use of research findings (Moher et al., 2010).

A strength of the RANCARE guideline is the development within the ongoing RANCARE COST Action, which allowed us to integrate new knowledge of the topic without the usual delays inherent in the publication process. Still, the sampling approach for the consensus process was oriented primarily toward objective criteria such as authorship of publications and was not limited only to the affiliation with the COST Action. This approach ensured raters' research experience and yielded a sample with a broad background.

The development of a reporting guideline requires an iterative approach, critical elements of which include a consensus process and, if possible, a Delphi survey (Moher et al., 2010). Therefore, as recommended for a Delphi survey, we defined consensus a priori in accordance with the RAND/UCLA Appropriateness Method (Diamond et al., 2014). Since the quantitative measurement was not sensitive enough in the first survey, we decided to add an additional question about inclusion of items with a stricter threshold (yes/no) for the second survey. As we were aiming for the most useful guideline possible for our target audience and based on the rich feedback received through both the surveys and the workshop, we decided to prioritize qualitative comments from participants if they highlighted potential for further improvement on clarity of the items. This has been previously described in the development of the ESPACOMP Medication Adherence Reporting Guideline (De Geest et al., 2018).

## 6 CHALLENGES AND LIMITATIONS

We faced several challenges in conducting this study. To address as much of the research on unfinished nursing care as possible we considered numerous forms for this guideline (e.g., self‐standing guideline or extension, targeting self‐report and other measures, focused on quantitative, qualitative, or mixed methods). As this guideline's aim is to facilitate comparison between studies, this primary iteration of the RANCARE guideline deals entirely with quantitative research, using either traditional self‐report survey methodology or novel data generation mechanisms like the electronic health record. Given the complexity of unfinished nursing care, we acknowledge the need to address qualitative study designs and would plan to address this limitation in future iterations. Still, with its overlap of items from other guidelines such as STROBE or CONSORT, this version can be widely applied for observational or interventional study designs. And while the full item set has been compiled to support detailed reporting on unfinished nursing care, items can be used individually if this phenomenon is treated as an antecedent or co‐variable.

Throughout the RANCARE guideline, we use *unfinished nursing care* as an umbrella term to ensure inclusion of all related concepts. While our inclusion of *nursing* within this term acknowledges nurses' crucial roles within health‐care delivery, we also acknowledge that healthcare is multidisciplinary and encourage researchers of other disciplines to use the RANCARE guideline. Rather than focusing on one specific concept or terminology, the RANCARE guideline can provide guidance to all research investigating unfinished, rationed, missed, omitted, compromised, or underused care (e.g., Glasziou et al., 2017).

## 7 CONCLUSION AND IMPLICATIONS

The RANCARE guideline's primary aim is to increase transparency and comprehensiveness in the reporting of quantitative studies on unfinished nursing care. More transparent and complete research findings are needed to better understand the link between unfinished nursing care and patient and/or nurse outcomes (Recio‐Saucedo et al., 2018; Vincelette et al., 2019). Researchers who follow the RANCARE reporting guideline will be able to communicate their research findings more clearly and comprehensively. This will allow more meaningful comparisons across studies, settings, and countries, thereby supporting more efficient use of empirical findings. More comprehensive, transparent and detailed reporting of estimates of unfinished nursing care will also help researchers and nurse managers target purposeful actions within their settings with the goal of improving the safety and quality of health‐care services. For further actions, we encourage researchers not only to follow the RANCARE guideline within publications but also to provide the authors with feedback on its usefulness.

## CONFLICT OF INTERESTS

The authors declare that there are no conflict of interests.

## ETHICS STATEMENT

In accordance with Swiss law, the responsible ethics committee exempted the Delphi study from its oversight (EKNZ)‐Req‐2018‐00399.

## APPENDIX A MEMEBERS OF THE RANCARE CONSORTIUM

RANCARE Consortium COST Action–CA 15208: Chair: Papastavrou Evridiki (Cyprus, Cyprus University of Technology); Vice Chair: Lemonidou Chryssoula (Greece, University of Athens); WG Leaders: Sermeus Walter (Belgium, Leuven Institute for Healthcare) Schubert Maria (Switzerland, University of Basel); Suhonen Riitta (Finland, University of Turku); Riklikiene Olga (Lithuania, Lithuanian University of Health Sciences); Acaroglu Rengin (Istanbul University, Turkey); Andreou Panayiota (Cyprus, Cyprus University of Technology); Antonic Darijana (Bosnia &Herzegoviva, Public Health Institute, Banja Luka, Republic of Srpska); Ausserhofer Dietmar (Italy Landesfachhochschule fur GesundheitsberufeClaudiana); Baret Christophe (France, CNRS, LEST); Bosch‐Leertouwer Helen (Netherlands, Windesheim University of Applied Sciences); Bragadottir Helga (Iceland, University of Iceland); Bruyneel Luk (Belgium, KatholiekeUniversiteit Leuven); Christiansen Karin (Denmark, VIA University College); Čiutienė Rūta (Lithuania, Kaunas University of Technology); Cordeiro Raul (Portugal, Instituto Politecnico de Portalegre); Deklava Liana (Latvia, Riga Stradins University); Dhaini Suzanne (Lebanon, American University of Beirut); Drach‐Zahavy Anat (Israel, University of Haifa); Eftathiou Georgios (Cyprus, Cyprus University of Technology); Ezra Sigal (Israel, Sheba Hospital, Sheba Medical Center); Pilan Fuster (Spain, UniversitatInternacional de Catalunya); Gotlib Joanna (Poland, Medical University of Warsaw); Gurkova Elena (Slovakia, University of Presov); Habermann Monika (Germany, Hochschule Bremen Neustadtswall); Halovsen Kristin (Norway, Oslo and Akershus University College Applied Sciences); Hamilton Patti (USA, Texas Woman's University); Harvey Clare (Australia, CQUniversity Australia); Hinno Saima (Estonia, Tartu Health Care College); Hjaltadottir Ingibjorg (Iceland, University of Iceland); Jarosova Darja (Czech Republic, University of Ostrava); Jones Terry (USA, Virginia Commonwealth University); Kane Raphaela (UK, Liverpool John Moore University); Kirwan Marcia (Ireland, Dublin City University, School of Nursing and Human Sciences); Leino‐Kilpi Helena (Finland, University of Turku); Leppée Marcel (Croatia, Institute for Healthy Ageing, Slovenska); Amorim Lopes Mario (Portugal, INESC‐TEC); Millere Inga (Latvia, Riga Stradins University); Ozsaban Aysel (Turkey, Istanbul University); Palese Alvisa, (Italy, Udine University); Patiraki Elisabeth (Greece, University of Athens); Pavloska Katina (FYR Macedonia, Institute for mental health for children and youth); Phelan Amanda (Ireland, University College Dublin, School of Nursing, Midwifery & Health Systems); Postolache Paraschiva (Romania, "Grigore T. Popa" University of Medicine and Pharmacy of Iasi); Prga Ivana (Croatia, Andrija Stampar Teaching Institute of Public Health); Rasch Agripina (Romania, University of Medicine and Pharmacy Carol Davila); Rengel Diaz Cristobal (Spain, Hospital Universitario Virgen de la Victoria de Malaga, Campus Universitario de Teatinos); Rochefort Christian (Canada, University of Sheebrook); Scott Anne (Ireland, National University of Ireland, Galway); Simon Michael (Switzerland, University of Basel); Stemmer Renate (Germany, Catholic University of Applied Sciences Mainz); Tichelaar Erna (Netherlands, Windesheim University of Applied Sciences); Toffoli Luisa (Australia, University of South Australia); Tonnessen Siri (Norway, University College of Southeast Norway); Uchmanowicz Izabella (Poland, Wroclaw Medical University); Vuckovic Jasminka (Bosnia & Herzegovina, Ministry of Health and Social Welfare Republic of Srpska); Willis Eileen (Australia, Flinders University); Xiao Lily (Australia, Flinders University); Zeleníková Renáta (Czech Republic, University of Ostrava); Zorcec Tatjana (FYR Macedonia, University Children's Hospital Faculty of Medicine University of Skopje).
